# An Alternatively Translated Connexin 43 Isoform, GJA1-11k, Localizes to the Nucleus and Can Inhibit Cell Cycle Progression

**DOI:** 10.3390/biom10030473

**Published:** 2020-03-20

**Authors:** Irina Epifantseva, Shaohua Xiao, Rachel E. Baum, André G. Kléber, TingTing Hong, Robin M. Shaw

**Affiliations:** 1 Smidt Heart Institute, Graduate Program in Biomedical Sciences, Cedars-Sinai Medical Center, Los Angeles, CA 90048, USA; iepifantseva@gmail.com (I.E.); sxiao@mednet.ucla.edu (S.X.); rachel.e.baum@utah.edu (R.E.B.); TingTing.Hong@cshs.org (T.H.); 2 Department of Pathology, Beth Israel & Deaconess Medical Center, Harvard Medical School, Boston, MA 02115, USA; akleber@bidmc.harvard.edu; 3 Department of Medicine, University of California Los Angeles, Los Angeles, CA 90048, USA; 4 Nora Eccles Harrison Cardiovascular Research and Training Institute, University of Utah, Salt Lake City, UT 84112, USA

**Keywords:** connexin43, trafficking, internal translation, cell cycle

## Abstract

Connexin 43 (Cx43) is a gap junction protein that assembles at the cell border to form intercellular gap junction (GJ) channels which allow for cell–cell communication by facilitating the rapid transmission of ions and other small molecules between adjacent cells. Non-canonical roles of Cx43, and specifically its C-terminal domain, have been identified in the regulation of Cx43 trafficking, mitochondrial preconditioning, cell proliferation, and tumor formation, yet the mechanisms are still being explored. It was recently identified that up to six truncated isoforms of Cx43 are endogenously produced via alternative translation from internal start codons in addition to full length Cx43, all from the same mRNA produced by the gene *GJA1*. GJA1-11k, the 11kDa alternatively translated isoform of Cx43, does not have a known role in the formation of gap junction channels, and little is known about its function. Here, we report that over expressed GJA1-11k, unlike the other five truncated isoforms, preferentially localizes to the nucleus in HEK293FT cells and suppresses cell growth by limiting cell cycle progression from the G_0_/G_1_ phase to the S phase. Furthermore, these functions are independent of the channel-forming full-length Cx43 isoform. Understanding the apparently unique role of GJA1-11k and its generation in cell cycle regulation may uncover a new target for affecting cell growth in multiple disease models.

## 1. Introduction.

Gap Junction (GJ) channels are formed by connexin proteins, which assemble as hexamers on the cell membrane and dock with the hexamer in an adjacent cell, creating a channel for intercellular communication [1]. Connexin 43 (Cx43) is the most prevalent gap junction protein in the heart and is widely expressed in most mammalian organs and cell types [2]. Dysregulation in Cx43 protein expression is associated with cardiac arrhythmias [3]. Studies have identified numerous non-canonical roles of Cx43 that are not described by gap junction channel activity, including in ischemic injury protection [4,5], cancer [6,7,8,9], wound healing [10], muscle differentiation [11], organ morphogenesis [12,13], and cell migration in embryonic development [14]. With specific regard to cancer, Cx43 can function as a tumor suppressor [6,15,16]. A reduction in Cx43 expression is observed in tumor cell lines [17], and reduced Cx43 has been proposed as a biomarker of malignant tissue [8,18,19,20]. Few studies have been able to reconcile how the gap junction Cx43 channel function can influence these apparently channel-independent roles of Cx43 protein.

Cx43 is encoded by the *GJA1* gene, which has a single coding exon [21]. Therefore, *GJA1* cannot provide protein diversity by means of alternative splicing of exons. However, it has recently been found that several small isoforms of Cx43 are produced by alternative translation, with methionines within the coding exon serving as internal translation start sites [22,23]. The alternatively translated small isoforms lack the N-terminal portions of Cx43 upstream of the initiating start sites, while retaining the remaining downstream C-terminus portion. There are six internal methionines and a total of six truncated protein isoforms can be produced from Cx43 mRNA with expression levels varying between isoforms and across cell types [22]. We have previously reported that the size of the most predominantly expressed isoform is 20kDa, thus termed GJA1-20k. It has been identified that GJA1-20k functions in the forward trafficking of full-length Cx43 to the plasma membrane to form gap junction channels [22,24] and is necessary for actin stabilization [24]. In addition to its role as a chaperone [22,24], GJA1-20k is upregulated in response to hypoxic [25] and oxidative stress for preventing mitochondrial fragmentation [26] and to mediate ischemia preconditioning protection [24,27]. Xenopus derived GJA1-20k has also recently been identified to localize to the cell nucleus, functioning as a transcription activator of N-Cadherin [28].

Nucleus localization of one or more of the smaller isoforms provides the opportunity to affect cell cycle. The overexpression of various portions of the C-terminus fragment of Cx43 (Cx43 CT) has been shown to occasionally localize to the nucleus [29,30] and is implicated in the suppression of cell proliferation [29,31,32]. Now that the six endogenous truncation isoforms have been identified [22], previous studies exploring various lengths of the C-terminus can be placed in the context of proteins that can be endogenously generated by alternative translation.

In this study, we perform an expression analysis of all six mammalian isoforms and make the surprising observation that only mammalian GJA1-11k, and not GJA1-20k or any of the other Cx43 isoforms, is preferentially enriched in the nucleus of mammalian cells. Moreover, we find that GJA1-11k can interfere with cell growth by limiting cell cycle progression in the G_0_/G_1_ phase.

## 2. Materials and Methods

### 2.1. Cell Culture

HEK293FT (Thermo Scientific) cells at low passages were grown on petri dishes at 37 °C in a humidified atmosphere with 5% CO2 in Dulbecco’s Modified Eagle’ Medium (DMEM, Thermo Scientific) high glucose with 10% fetal bovine serum (FBS), nonessential amino acids, sodium pyruvate (Thermo Scientific), and antibiotics Mycozap-CL (Lonza). For the expression of targets, cells were plated down 24h prior to transient transfection at a density indicated in each experiment. Transient transfection was carried out using either Lipofectamine^TM^ 2000 (Thermo Scientific) or FuGENE® HD (Promega). Transfection by both reagents consistently resulted in 75%–80% transfection efficiency, assessed by Flow Cytometry. The cells were used for assay after 48 hours of transfection unless otherwise stated in the figure legends.

### 2.2. RNAi Interference

Chemically synthesized siRNAs (Thermo Scientific) to knockdown *GJA1* mRNA (sequence 5’ to 3’: GG GAG AUG AGC AGU CUG CCU UUC GU; HSS178257) and Stealth^TM^ RNAi with medium GC content as a negative control (scramble, cat. # 12935-112) were used. HEK293FT cells at the density 2 × 10^6^ were transfected with 100nM of either siRNA or the scramble control according to the manufacturer’s reverse transfection protocol using Lipofectamine^TM^ RNAiMAX (Thermo Scientific). The cells were manually counted at 24, 48, 72, 96, and 120 h after transfection.

### 2.3. Molecular Biology

Human *GJA1* encoding full-length and small isoforms were obtained from Open Biosystems and cloned into pDONR/221 using Gateway BP cloning to generate entry clones (BP clonase II; Thermo Scientific). Destination vectors (pDEST) encoding C-terminal V5-, HA-tagged and C-terminal NES (nuclear export signal) V5-tagged proteins were subsequently made using entry clones and Gateway LR cloning (LR clonase; Thermo Scientific). All constructs for mammalian cell expression are driven by the cytomegalovirus (CMV) promoter and include downstream internal mutated methionine-to-leucine start sites of Cx43 to ensure single isoform expression. Mutagenesis was carried out with QuickChange Lightning Mutagenesis Kit (Agilent) according to the manufacturer’s protocol.

### 2.4. Immunofluorescence Staining

HEK293FT cells were plated on 35-mm fibronectin-coated glass bottom culture dishes (MatTek Corporation, cat #P35G-1.0-14-C) 24 hours before transfection. The cells were transfected with 2mg of two plasmids—the negative control pcDNA3.2-GW-CAT-V5 and the positive control pcDNA3.2-GFP-V5—to verify the transfection efficiency, wild type pcDNA-GJA1-WT-V5, and all of the alternatively translated isoforms of the Cx43 protein (pcDNA-GJA1-43k-V5, pcDNA-GJA1-32k-V5, pcDNA-GJA1-29k-V5, pcDNA-GJA1-26k-V5, pcDNA-GJA1-20k-V5, pcDNA-GJA1-11k-V5, pc DNA-GJA1-7k-V5) and pcDNA-GJA1-11k-NES-V5. At 36h post transfection, the cells were washed in PBS and fixed in 4% paraformaldehyde for 15 min at RT. The cells were permeabilized in 0.1% Triton in PBS and blocked in 5% goat serum for 1 h and were then subsequently incubated with primary antibodies for 3 h at RT: rabbit polyclonal anti-Cx43, raised against a 17-residue peptide from the C-tail of Cx43 (1:1000, Sigma), and mouse monoclonal anti-V5 (1:50, Sigma). After washing 3 times with 0.1% Tween-20 in PBS, the cells were incubated with secondary antibodies: goat anti-mouse IgG conjugated to Alexa Fluor 488 (1:500, Thermo Scientific) and goat anti-rabbit IgG conjugated to Alexa Fluor 555 (1:500, Thermo Scientific) for 1 h at RT. The nuclei were detected by Hoechst 33342, trihydrochloride, trihydrate (1µg/ml, Thermo Scientific) staining for 5 min at RT and were then mounted to ProLong Gold Antifade reagent (Thermo Scientific).

### 2.5. Image Processing

Images were taken using a Nikon Eclipse T*i* microscope with a 100×/0.75 Plan Apo objective and a Yokogowa CSU-X1 spinning-disk confocal unit with 350, 486, 561-laser units, and an ORCA-Flash 4.0 Hamamatsu camera (C11440), controlled by NIS Elements software and analyzed using Adobe Photoshop. The HEK293FT cells were imaged at z-depth increments of 0.3 μm. One plane image was used for quantifications in ImageJ (National Institute of Health, Bethesda, MS, USA) and the focused image (maximum projection intensity) of z-stacks was used for the publication. Fluorescence intensity profiles were generated and quantified by Image J in at least 20 cells per sample. The ratio of nuclear/cytoplasmic fluorescence intensity of Cx43 small isoforms normalized to the region of interest (ROI) detected by both antibodies (anti-V5 and anti-Cx43) was quantified. Each image was background-subtracted using a rolling ball radius of 50 pixels.

### 2.6. Cell Count and Cell Cycle Assay

The cells were plated down at the density 2 × 10^6^ cells per 100mm petri dish and were exposed to serum starvation in complete media with 0.2% FBS for 48 h to ensure maximum cell cycle synchronization without cell cycle arrest. Then, the cells were transfected in 10% serum-supplemented media with various plasmids of interest using FuGENE® HD (Promega) according to manufacturer’s protocol. The concentration of plasmid cDNA of each sample was normalized to the amount of protein produced in given cells in 48 h measured by the Western Blot signal intensity normalized to actin.

On 1st and 2nd days after transfection, the cells were trypsinized and manually counted. On day 3 after transfection, the cells were incubated with 10 µM BrdU (37 °C, 45min in complete media) and were collected for cell cycle assay and manual count. Then, the cells were fixed and stained with anti-BrdU antibodies and 7-AAD DNA dye according to FITC BrdU flow kit (BD Pharmingen^TM^) manual instruction. The cells were excited at 488nm, and signals from 50,000 cells were acquired at 585/42 and 702/64 in LSR II (BD Biosciences). The results were analyzed using FlowJo software and were expressed as the percentage of cells in each cell cycle phase within the entire population excluding debris and apoptotic cells. At least triplicates were used for each sample and each experiment was conducted four times.

### 2.7. Western Blot and Subcellular Fractionation

To detect protein expression by Western Blotting, the cells were plated down in 6-well plates at a concentration 0.65 × 10^6^ per well. The next day, the cells were transfected with plasmid cDNA using Lipofectamine^TM^ 2000 (Thermo Scientific) according to the manufacturer’s instructions. The cells were lysed in RIPA buffer containing (mM): 0.1%SDS, 50 mM Tris, pH 7.4, 150mM NaCl, 1mM EDTA, 1% Triton X-100, 1% sodium deoxycholate, 1 mM NaF, 200µM Na_3_VO_4_, and 1× Halt Protease and Phosphatase Inhibitor Cocktail (Thermo Scientific), and were ruptured by sonication before centrifugation at 10,000 × g for 20 min at 4 °C. The cell lysates were normalized for total protein with BCA assay (Bio-Rad DC Protein Assay). The samples with 4× NuPage sample buffer (Thermo Scientific) supplemented with dithiothreitol (DTT, 400mM) were heated at 70 °C for 5 min, cooled to RT, and 100µg per lane were separated by NuPAGE Bis-Tris 4%–12% gradient gel (Thermo Scientific) in MES running buffer (Thermo Scientific). The gels were transferred in 10% Methanol-containing transfer buffer to FluoroTrans PVDF membranes (Pall), which were subsequently blocked in 5% non-fat milk (Carnation) in TNT buffer (0.1% Tween-20, 150 mM NaCl, 50mM Tris pH 8.0) for 1 h at RT. Membranes were probed overnight with primary antibodies diluted in 5% milk in TNT. Mouse monoclonal anti-Cx43 directed against C-terminal region, prepared to the last 23 amino acids of Cx43 (1:500, Millipore), mouse monoclonal anti-β-actin (1:2000, Sigma-Aldrich), mouse monoclonal anti-V5 (1:500, Sigma-Aldrich), and goat secondary antibodies conjugated to AlexaFluor 647 (1:500, Thermo Scientific) were used in this study. The membranes were imaged with a ChemiDocMP4000 fluorescent western detection system (BioRad). The membranes were stripped using Re-Blot plus Strong solution (Millipore) and re-probed with β-actin to ensure equal loading and were used as a normalization control. The relative intensity of signals was quantified using BioRad software.

The Nuclear Extraction kit (ab113474, Abcam) was used to obtain nuclei, and cytosolic-enriched fractions from HEK293FT cells transfected with GJA1-11k, GJA1-43k, cDNA plasmid, or negative control GFP-V5 cDNA. After reconstitution in RIPA buffer, fractions were analyzed by Western Blot in Bis-Tris 10% SDS-PAGE gel (100µg protein/lane), transferred to PVDF membrane and probe to mouse monoclonal anti-Cx43 directed against the C-terminal region (1:500, Millipore), mouse monoclonal anti-V5 (1:200, Sigma-Aldrich). To confirm the enrichment of correct proteins in obtained fractions, the same blot was probed with nuclear marker mouse monoclonal anti-Histone H3 (1:2000, Abcam), cytoplasmic marker mouse monoclonal anti-GAPDH (1:5000, Abcam), membrane marker mouse monoclonal NA+/K+-ATPase (1:10000, Millipore), Golgi marker rabbit monoclonal anti-GMP130 (1:1000, Abcam), and nuclear envelope marker rabbit monoclonal anti-Lamin B1 (1:5000, Abcam).

### 2.8. Statistical Analysis

All quantitative data are presented as mean ± SEM, with ‘n’ denoting of the number of independent experiments. The normality of the data sets was tested using Kolmogorov-Smirnov’s test and d’Agostino and Pearson’s test. For comparison between the two groups, an unpaired two-tail Student’s *t*-test was performed. For comparison among three or more groups, one-way ANOVA (followed by Tukey’s post-hoc test) or two-way ANOVA (followed by Tukey’s multiple comparison test) were used accordingly and analyzed in Prism 6 software (GraphPad). A *p*-value < 0.05 was considered significant.

## 3. Results

### 3.1. Alternatively Translated Cx43 Isoforms Localize to Different Subcellular Compartments

We constructed separate cDNA plasmids, each expressing an alternatively translated isoform that can be generated from internal methionine start sites (GJA1-32k, GJA1-29k, GJA1-26k, GJA1-20k, GJA1-11k, GJA1-7k). For each of the plasmids, the AUGs downstream of each start codon were replaced by CUGs so that each plasmid only produces one Cx43 protein isoform fused with a C-terminal V5-tag. We also constructed a wild-type Cx43 (GJA1-WT) plasmid with all internal AUG start sites intact and a plasmid encoding only full-length Cx43 (GJA1-43k), which preserved the first start codon yet with each downstream AUG mutated to CUG (Figure S1). Human embryonic kidney HEK293FT cells have little endogenous Cx43 and therefore present little endogenous competition to the exogenous overexpression of Cx43 isoforms.

**Figure 1:**
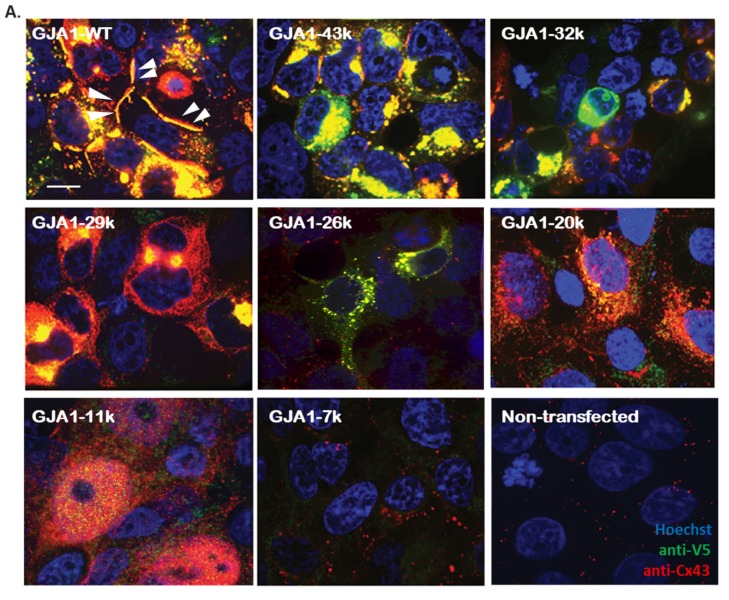

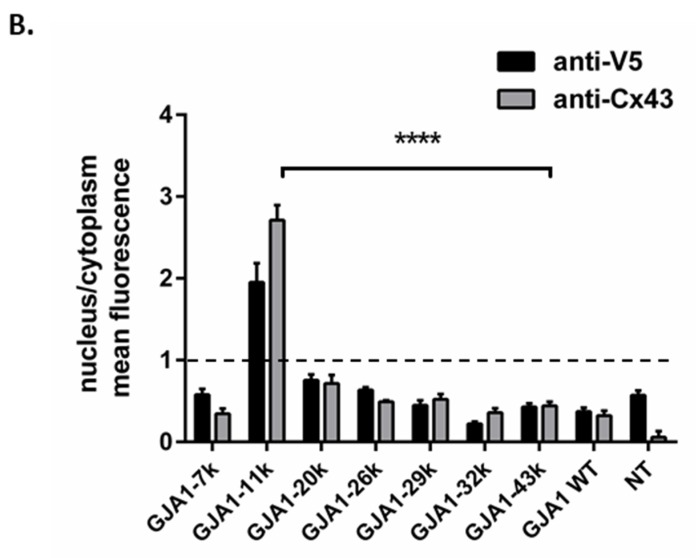
Localization of alternatively translated Cx43 isoforms expressed in HEK293FT cells. (**A**). The fixed-cell immunofluorescence of HEK293FT cells expressing short isoforms of Cx43 was detected by monoclonal anti-V5 (green) and polyclonal anti-Cx43 (red) antibodies, raised against a 17-residue peptide of C-terminal Cx43 (Sigma) with Hoechst staining of the nuclei (blue). The arrow heads show the gap junction (plaque) formation at the cell–cell border of cells with wild type (GJA1-WT) plasmid. Scale bar: 10μm. The results are representative of four independent experiments. (**B**). The ratio of fluorescent intensity in the nucleus versus cytoplasm in transfected HEK293FT cells. The data are presented as mean ± SEM, n = 20, **** *p* < 0.0001, by two-way ANOVA followed by Tukey’s multiple comparison test. The intensities were measured and normalized to background using Image J.

Using immunofluorescence, we used two separate antibodies (anti-Cx43 C-terminus and anti-V5) to identify the subcellular localization of each V5-tagged isoform when exogenously introduced in HEK293FT cells (Figure 1A). As expected, GJA1-WT localized as large plaque formations on the cell–cell border. In contrast, full length GJA1-43k accumulated in perinuclear regions, indicating a reduced ability to be trafficked to the cell border, consistent with our previously reported results of a six-fold reduction in gap junction plaques [22]. GJA1-20k, a chaperone that directs the trafficking of the full-length GJA1-43k isoform to the cell–cell border, generally appears localized to the perinuclear and ER regions and the mitochondria [26]. Of note, we did not detect significant GJA1-20k localization to the cell nucleus. However, GJA1-11k appeared to be highly enriched in the nucleus, with minimal detection in the cytoplasm.In general, V5 detection is diminished as the isoforms get smaller, yet the nucleus to cytoplasm ratios remained consistent. GJA1-7k, the smallest isoform, was poorly detected by either antibody. We also stained non-transfected cells to establish the baseline endogenous expression of Cx43 in HEK293FT cells.

To quantify the distribution of each isoform, we calculated the ratio of the mean fluorescent density in the nucleus versus cytoplasm (Figure 1B). This analysis confirmed that GJA1-11k has a two (anti-V5 antibody detection) to threefold (anti-Cx43 detection) nuclear enrichment. Furthermore, the nuclear localization is specific to GJA1-11k. All other isoforms capable of being generated by alternative translation had 50% or less nucleus to cytoplasm density. To confirm that nucleus localization was not secondary to the V5 tag interacting with GJA1-11k, we obtained similar results by a staining for HA-tagged GJA1-11k (Figure S2).

**Figure 2:**
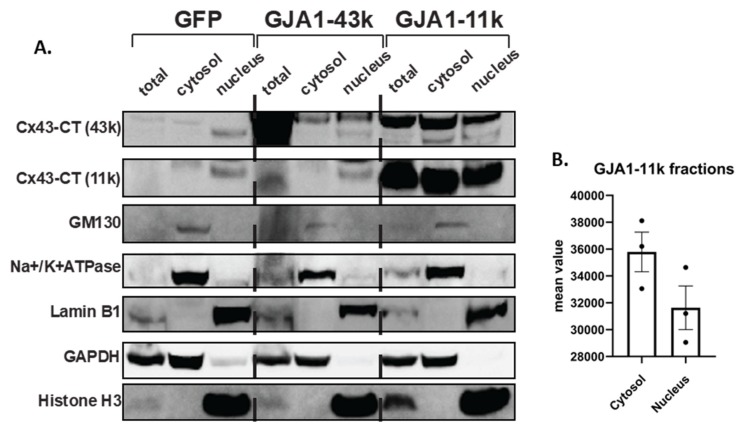
(**A**). Biochemical isolation of GJA1-11k in the nucleus. Western Blot of Cx43 isoforms in subcellular fractions (cytosolic and nuclear fractions) of HEK293FT cells over-expressing exogenous GJA1-11k, GJA1-43k, and GFP-V5 tagged plasmid cDNA, probed to monoclonal Cx43-CT (Millipore) antibodies. To demonstrate enriched biochemical isolation, the same blot was probed using antibodies to different subcellular fraction markers, including cytoplasmic (GAPDH), membrane (Na^+^/K^+^-ATPase), Golgi (GM-130), and nuclear markers (Histone H3, Lamin B1). The results are representative of three independent experiments. (**B**). The densitometry of CT-43k (11k) subcellular fractions assessed by Western Blot. Uncut immunoblots are provided in Figure S3.

The nucleus localization of GJA1-11k was also tested by biochemistry techniques. We performed subcellular fractionations of HEK293FT cells overexpressing GJA1-11k, GJA1-43k, and a negative control GFP-V5. Consistent with the immunofluorescence data, Western Blot analysis of cytosolic and nuclear fractions revealed that when compared to full length GJA1-43k, GJA1-11k expression is more enriched in the nucleus, with nearly as much protein in the nucleus as in the cytoplasm despite a much smaller intranuclear volume (Figure 2, Figure S3). Together, the data of Figure 1 and Figure 2 demonstrate that a major fraction of GJA1-11k, unlike the other mammalian Cx43 isoforms, localizes to the nucleus.

### 3.2. GJA1-11k Inhibits Cell Proliferation

**Figure 3:**
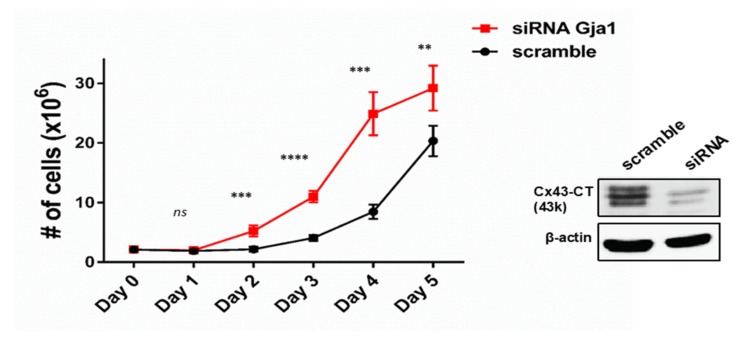
Effect of Cx43 on cell proliferation. The knockdown of endogenous Cx43 increases the cell proliferation of HEK293FT cells. For each day, data are presented as mean ± SEM, *n* = 9, ** *p* < 0.01, *** *p* < 0.001, **** *p* < 0.0001 by an unpaired two-tail Student’s *t* test. Western Blot analysis confirmed the knockdown of Cx43 and is representative of three independent experiments.

The nucleus is the primary organelle involved in cell cycle regulation. Because the C-terminus fragments are associated with inhibiting cell proliferation [29,31] and GJA1-11k appears to be enriched in the nucleus (Figure 1 and Figure 2), we quantified cell proliferation in the presence of GJA1-11k. HEK293FT cells were transfected with the siRNA that silenced endogenous GJA1 mRNA, removing any endogenous source of any Cx43 isoform. Note that silencing GJA1 alone, even with little background signal, resulted in a significant increase in cell growth compared to non-transfected cells or scramble siRNA control (Figure 3, Figure S4). These data suggest that even minute quantities of Cx43 or its isoforms can inhibit cell proliferation.

**Figure 4:**
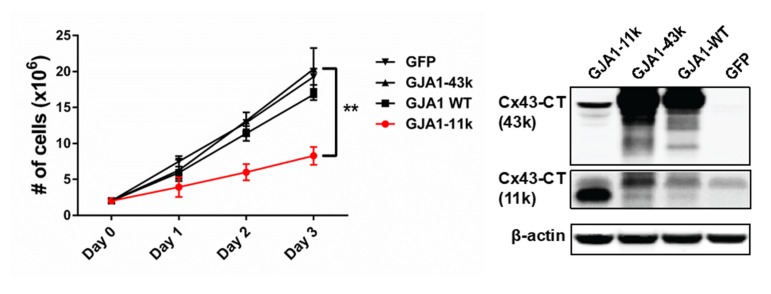
Effect of Cx43 short isoforms on cell proliferation. The overexpression of GJA1-11k inhibits the cell growth of the HEK293FT cell line. The growth curve is counts of transiently transfected cells each with vectors expressing GJA1-11k, GJA1-43k, GJA1-WT, and the negative control plasmid GFP with V5-tag. For each day data are presented as mean ± SEM, *n* = 12, ** *p* < 0.01 by one-way ANOVA followed by Tukey’s post-hoc test. Cell growth analysis revealed that cells expressing GJA1-11k grew significantly slower, than cells overexpressing other isoforms. Western Blot confirmed transient expression of Cx43 isoforms. Uncut immunoblots are provided in Figure S6.

We then explored the effect of the full length Cx43 and the GJA1-11k isoforms on cell proliferation. We transfected HEK293FT with vectors expressing GJA1-11k, GJA1-43k, GJA1-WT, and GFP-V5 as a negative control. The cells were counted over a three-day period (Figure 4). GJA1-11k significantly inhibited cell growth (*p* < 0.01), relative to GJA1-43k and GFP-V5. Apoptosis was ruled out by TUNEL staining (Figure S5). Together, these results indicate that GJA1-11k can be a potent growth suppressor, more so than either the full-length isoform GJA1-43k or the intact WT Cx43.

**Figure 5:**
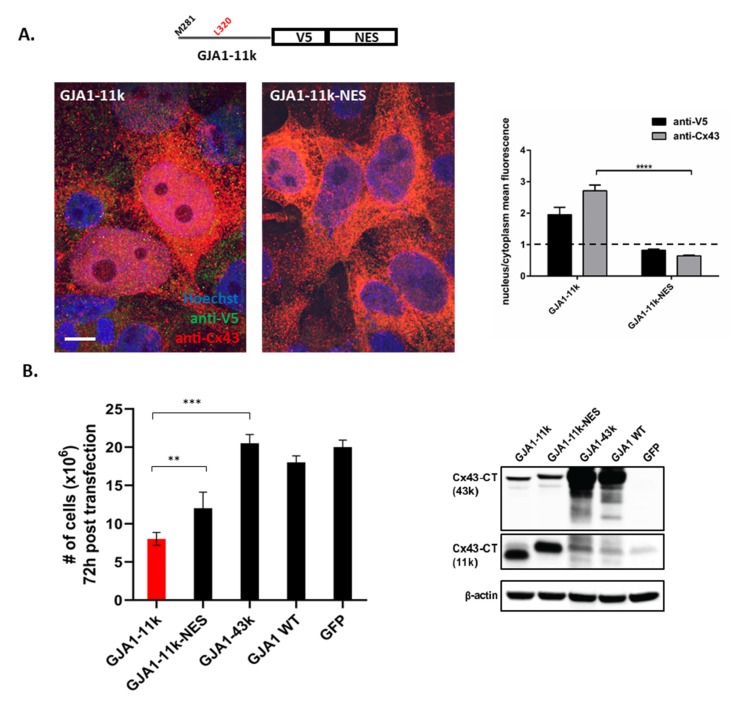
GJA1-11k with nuclear export signal rescues the cell proliferation of the HEK293FT cell line. (**A**). The immunofluorescence of HEK293FT cells expressing GJA1-11k or GJA1-11k-NES probed with V5 and Cx43 antibodies (Sigma) and the corresponding ratio of fluorescence density in the nucleus versus cytoplasm is shown on the graph. Scale bar: 10μm. The graph results are representative of four independent experiments. The data are presented as mean ± SEM, n = 26, **** *p* < 0.0001 by an unpaired two-tail Student’s *t* test. The intensities in the bar graph were measured and normalized to background using Image J. (**B**). GJA1-11k-NES rescues cell proliferation, suggesting that the effect of GJA1-11k is linked to the nucleus. The number of cells transiently transfected with vectors expressing GJA1-11k, GJA1-11k-NES, GJA1-43k, GJA1-WT, and the negative control plasmid GFP were counted 72 h after transfection. Cell growth analysis has shown that cells expressing GJA1-11k-NES grew significantly faster than cells expressing GJA1-11k. The Western Blot confirmed the transient expression of Cx43 isoforms. The data are presented as mean ± SEM, *n* = 12, ** *p* < 0.01, *** *p* < 0.001 by one-way ANOVA followed by Tukey’s post-hoc test.

We next tested whether nuclear localization is essential for GJA1-11k to convey growth inhibition. From an in silico analysis, we could not find a nuclear localization sequence (NLS) in GJA1-11k to remove and therefore limit its entry. However, we were able to add a nuclear export signal (NES) [33] to GJA1-11k. A vector was generated with an NES sequence (LQLPPLERLTLD) [34] added to GJA1-11k-V5, and transiently transfected into HEK293FT. We then used immunofluorescence to identify changes in nuclear localization. The expression of GJA1-11k-NES significantly reduced the presence of GJA1-11k in the nucleus (Figure 5A), *p* < 0.0001, whether detection was by antibodies against Cx43-CT or V5 tag (Figure 5A, right Panel), *p* < 0.001. We next quantified cell growth as done in Figure 4. The results show that GJA1-11k-NES partially rescues cell growth (Figure 5B, Figure S6). Residual decreases in cell count were likely due to residual GJA1-11k remaining in the nucleus.

### 3.3. GJA1-11k Inhibits Cell Cycle Progression to the S Phase

**Figure 6:**
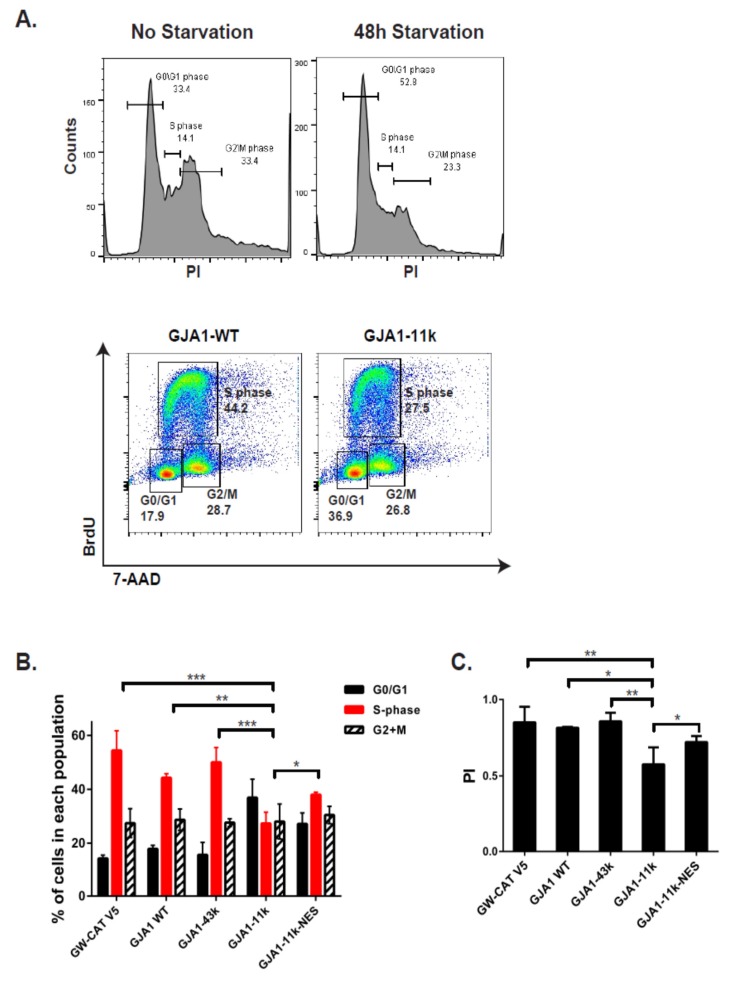
Overexpression of wild type Cx43 and short isoforms inhibits cell cycle progression in HEK293FT cells. (**A**). Non-transfected cells were serum-deprived for 48 h to synchronize cells in the G_0_/G_1_ phase. A representative image showing cell cycle phases in the HEK293FT cell line based on BrdU incorporation versus total DNA staining by 7-AAD. The cell cycle phases were analyzed by FlowJo. (**B**). The cell cycle distribution of GJA1-WT, GJA1-43k, GJA1-11k, and GJA1-11k NES V5-tagged isoforms were analyzed 72 h post-transfection. The bar graph shows the reduction in the number of cells in the S-phase and accumulation of cells in G_0_/G_1_ expressing GJA1-11k compare to GJA1-WT or GJA1-43k. GJA1-11k-NES rescues cell cycle progression from G_0_/G_1_ to S-phase. The data are presented as mean ± SEM, *n* = 12, * *p* < 0.05 and ** *p* < 0.01, *** *p* < 0.001 by one-way ANOVA followed by Tukey’s post-hoc test. (**C**). The Proliferation index (using following PI = ((S+G_2_/M) / (G_0_/G_1_+S+G_2_/M)) formula) confirmed the reduction in proliferation rate for GJA1-11k. The data are presented as mean ± SEM, *n* = 12, * *p* < 0.05 and ** *p* < 0.01, by one-way ANOVA followed by Tukey’s post-hoc test.

The inhibition of proliferation implies interruption of cell cycle progression. We explored the stage at which cell growth is inhibited by GJA1-11k in the nucleus. HEK293FT cells were serum-deprived for 48h to synchronize cells in the phase G_0_/G_1_ prior to transfection. Seventy-two hours after transfection, the cells were labeled with BrdU for 45 min and subsequently fixed and stained with anti-BrdU antibody (protocol diagrammed in Figure S7). Flow cytometry was used to quantify the percentage of cells in each phase (Figure 6A). Quantification was performed in cells transfected with either a negative control (GW-CAT V5), GJA1-WT, full length GJA1-43k, GJA1-11k, or GJA1-11k-NES. Our results in Figure 6B indicate that, of all the plasmids, GJA1-11k resulted in the highest percentage of cells remaining in G0/G1 (from 17% in the negative control to almost 40% with GJA1-11k, a twofold increase). As would be expected by capturing cells in G0/G1, the expression of GJA1-11k results in half of the cells in the S phase as it does in the negative control cohort (from 50% to 27%, *p* < 0.05; Figure 6B). The expression of GJA1-11k-NES resulted in increasing the percentage of cells in the S phase relative to GJA1-11k (*p* < 0.01; Figure 6B).

The flow cytometry data in Figure 6B can be used to quantify growth fraction by a proliferation index (PI). Of all the plasmids, only GJA1-11k significantly reduced the calculated PI (Figure 6C). Notably, the addition of the NES to GJA1-11k reverses the GJA1-11k induced reduction in PI (Figure 6C). Together, the results confirm a GJA1-11k induced reduction in the proliferation that occurs by limiting the exit of the G0/G1 phase and that nucleus localization is necessary to affect the growth inhibition.

### 3.4. Discussion

Our work attributes a specific biological role to mammalian GJA1-11k. The 11kDa alternatively translated isoform of Cx43 functions independent of gap junction formation as it uniquely localizes to the nucleus where it directly affects cell growth, limiting cell cycle progression from the G0/G1 phase to the S phase.

In our expression analysis (Figure 1), we found mammalian GJA1-20k to be distributed in cytoplasmic organelles, consistent with its known roles in Cx43 forward trafficking [22,24] and metabolism [26,27]. This reported localization differs somewhat to a recently published study about the role of Xenopus GJA1-20k, which was found to translocate to the nucleus of Xenopus and HeLa cells (where it can upregulate N-cadherin expression [28]). It is not clear why we could not reproduce Kotini et al’s [28] finding regarding GJA1-20k nucleus enrichment. The Xenopus GJA1-20k has a different sequence and is shorter than human GJA1-20k by six amino acids. The Xenopus plasmid also did not mutate downstream methionine resulting in potential generation of alternative isoforms GJA1-11kDa and 7kDa, which may have been detected in lieu of GJA1-20k. The Xenopus GJA1-20k in the Kotini study [28] was also covalently bound to the glucocorticoid receptor to enforce nuclear translocation of GJA1-20k, and thus nuclear localization could have been more a product of the glucocorticoid receptor that of the GJA1-20k itself. Finally, differences between the human and Xenopus GJA1-20k isoform sequences could result in differences in localization tendencies. Future studies carefully exploiting the differences in the plasmids and their effect on localization should be highly informative.

Cx43 expression is typically reduced in tumor cell lines [17] or tissues [8,18,19,20,35,36,37,38] and the loss of Cx43 is associated with shorter patient survival [8,20]. It has already been identified that ectopic expression of WT Cx43 in cancer cells does not result in gap junctions generation yet limits cell proliferation [7,8,39,40]. Despite these observations and correlations, significant knowledge gaps exist in our mechanistic understanding of non-canonical effects of ectopic Cx43. It should also be noted that most studies identify Cx43 by antibody staining in which the epitope for Cx43 specific antibodies is in the C-terminus; therefore, full length Cx43 cannot be differentiated from the smaller isoforms [22]. We expect that reports of Cx43 in the nucleus in cancer cells [28,29,30,31] indeed may be GJA-11k, which is protective against cancer progression. For exogenous introduction, GJA1-11k is a small, hydrophilic, nucleus-targeted peptide that could be exploited for cancer therapy. Future studies should explore the molecular mechanisms underlying nuclear, GJA1-11k-regulated cell cycle progression and cell growth, as well as its translation into a new therapy targeting tumor growth and progression. In this particular study, we used an in vitro model with only HEK cells which have fast growth. In addition, we relied on exogenous expression that can result in super-physiologic levels of transfected proteins. Finally, HEK cells have limitations in microscopy that can be overcome with flatter cells. Future studies should include multiple cell lines including cancer cell lines, as well as in vivo models.

Given the identified roles of GJA1-20k in channel trafficking [22,24], metabolism [25,26,27,41], transcription [28], as well as in epithelial-mesenchymal transition [42], we were surprised in this study that the dominant phenotype of GJA1-11k relates to yet another phenomenon, specifically inhibiting cell cycle progression. The common denominator, we believe, is the affinity of the Cx43 C-terminus for both microtubule [26] and actin [24] cytoskeletons. A microtubule-binding domain for Cx43 has been identified in the proximal Cx43 C-terminus [43,44]. If actin is involved in GJA1-11k nuclear localization, then the results of the current study would support early studies identifying an association between Cx43 and actin [24,45] and, in general, help us understand that cytoskeletal interactions are the basis for multiple fundamental properties of the internally translated isoforms of Cx43.

Our focus on cell cycle regulation was based on a combination of the classic role of proteins with intranuclear enrichment and prior reports that Cx43 involvement in cell-cycle regulation is not easily explained by sarcolemmal localized ion channels. As the field explores the effects of Cx43’s smaller isoforms, we expect the number of cellular phenomena associated with each isoform to grow and we will identify crossover between the roles of different isoforms. For instance, in unpublished studies, we observed that GJA1-11k can affect the transcription of GJA1 mRNA. Already, GJA1-20k is associated with the transcriptional regulation of N-Cadherin [28]. Similarly, proteins that rearrange the extranuclear cytoskeleton, such as GJA1-20k [24], may also affect the mechanics of cell proliferation. We look forward to the next several years of discovering the roles of Cx43’s smaller isoforms.

Formation of smaller truncated isoforms by alternative translation can be a source of considerable biologic diversity for any gene, and especially single coding exon genes such as Gja1 that are not able to undergo splicing. Traditionally, alternative translation is considered a phenomenon that occurs with development and evolutionary changes [46]. However, it has already been established that the expression of GJA1-20k and other smaller isoforms increase with metabolic stress [25,27] or as a result of mTOR and Mnk1/2 pathway inhibition. These pathways are involved in critical and dynamic cellular processes, such as cell growth, proliferation, transcription, and survival [22,23], and occur in mature cells on a much more acute time scale than during development and evolutionary change. Previous findings have identified GJA1-20k as a protective stress response protein in terminally differentiated cardiomyocytes [27]. Based on our results, GJA1-11k could also be a stress response protein, but its upregulation could be a response to inhibit cell cycle progression.

## Supplementary Materials

The following are available online at https://www.mdpi.com/2218-273X/10/3/473/s1, Figure S1: title, Table S1: title, Video S1: title.
