# Supplementary material for: An Alternatively Translated Connexin 43 Isoform, GJA1-11k, Localizes to the Nucleus and Can Inhibit Cell Cycle Progression
Source: Biomolecules. 2020 Mar 20;10(3):473. doi: 10.3390/biom10030473 (PMC7175147; doi:10.3390/biom10030473)
Supplement: Supplementary file 1 [file biomolecules-10-00473-s001.pdf]

Supplemental Figure 1

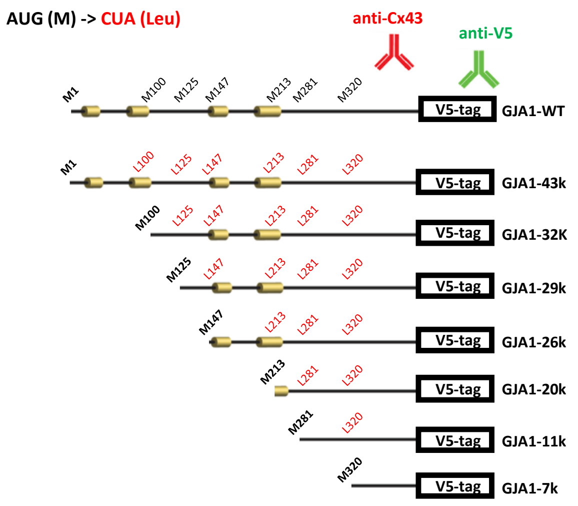

Figure 1S: Generation of Cx43 isoform constructs. The illustration represents cDNA plasmids coding different isoforms which vary in size and include wild type (WT, GJA1-WT) and alternatively translated isoforms of GJA1 (GJA1-43k, GJA1-32k, GJA1-29k, GJA1-26k, GJA1-20k, GJA1-11k, GJA1-7k) with a V5-tag added to the C-terminal region of the protein. This allows the ectopically expressed protein to be detected by anti-V5 monoclonal antibodies. While the GJA1-WT cDNA construct is able to express all seven protein isoforms (as all AUG start sites remains intact), expression of full-length protein (GJA1-43k) was generated by mutating all downstream Methionine start codons (AUG) to Leucine (CUA).

Supplemental Figure 2

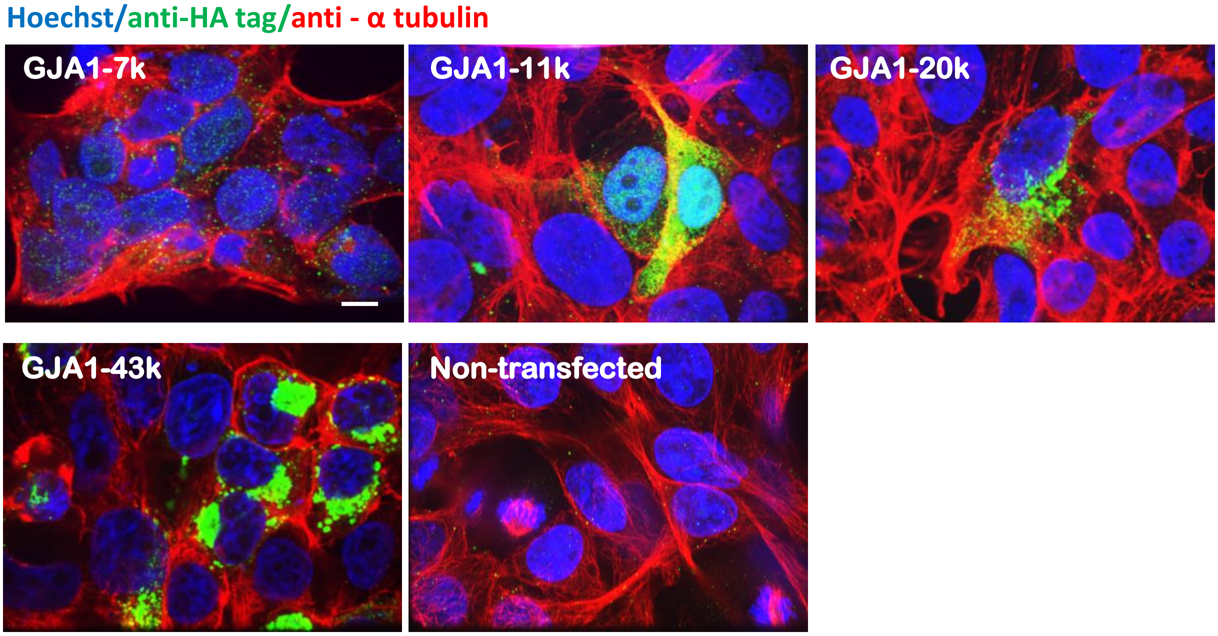

Figure S2: Smaller isoforms localization in HEK293FT cells. HA-tagged Cx43 isoforms showed the same pattern of localization. GJA1-11k with HA tag was observed in the nucleus. Scale bar: 10μm.

Supplemental figure 3

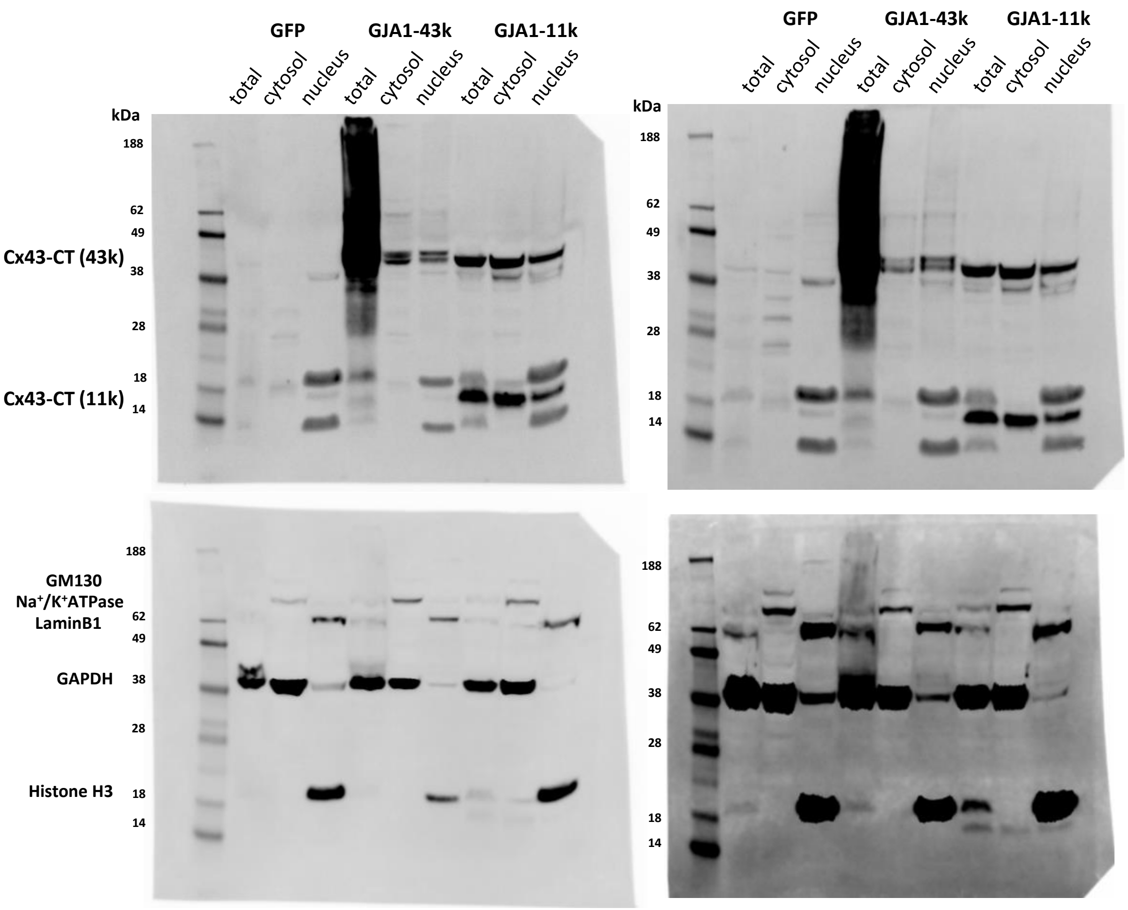

Figure S3: Uncut western blots probed to Cx43 antibodies (first raw) and markers of subcellular fractionations (second raw) for Figure 2.

Supplemental figure 4

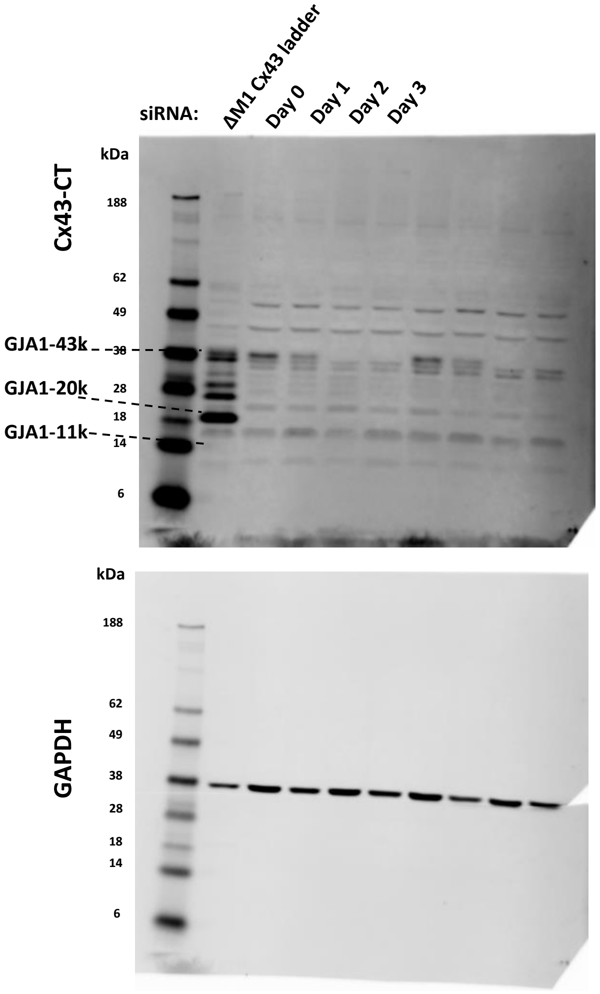

Figure S4: Uncut immunoblot of Cx43 siRNA silencing at day 0 (no silence), day 1, 2, 3 for Figure 3

Supplemental Figure 5

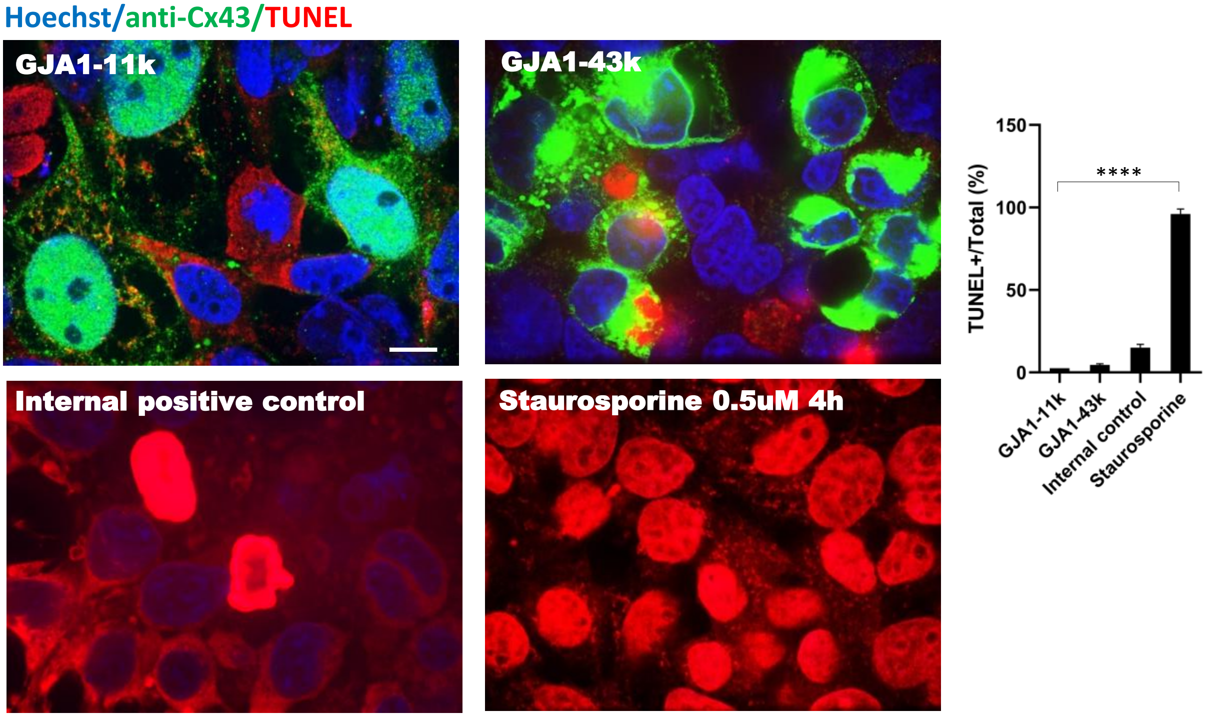

Figure S5: Overexpression of GJA1-11k does not induce apoptosis in HEK293FT cells. Effect of Cx43 short isoforms on cell proliferation is not due to apoptosis. Cells were immunostained after 72 h post-transfection to assess cell death using TUNEL (red) staining. Graph represents percentage of TUNEL-positive cells (red) normalized to total Hoechst-positive (blue) in cells overexpressing GJA1-11k and GJA1-43k (green) or treated with apoptosis-induced agent Staurosporine for 4h at 0.5uM. Data are presented as mean ±SEM, n=110, ****$p < 0.0001$ by one-way ANOVA followed by Tukey's post-hoc test. Scale bar: 10µm.

Supplemental figure 6

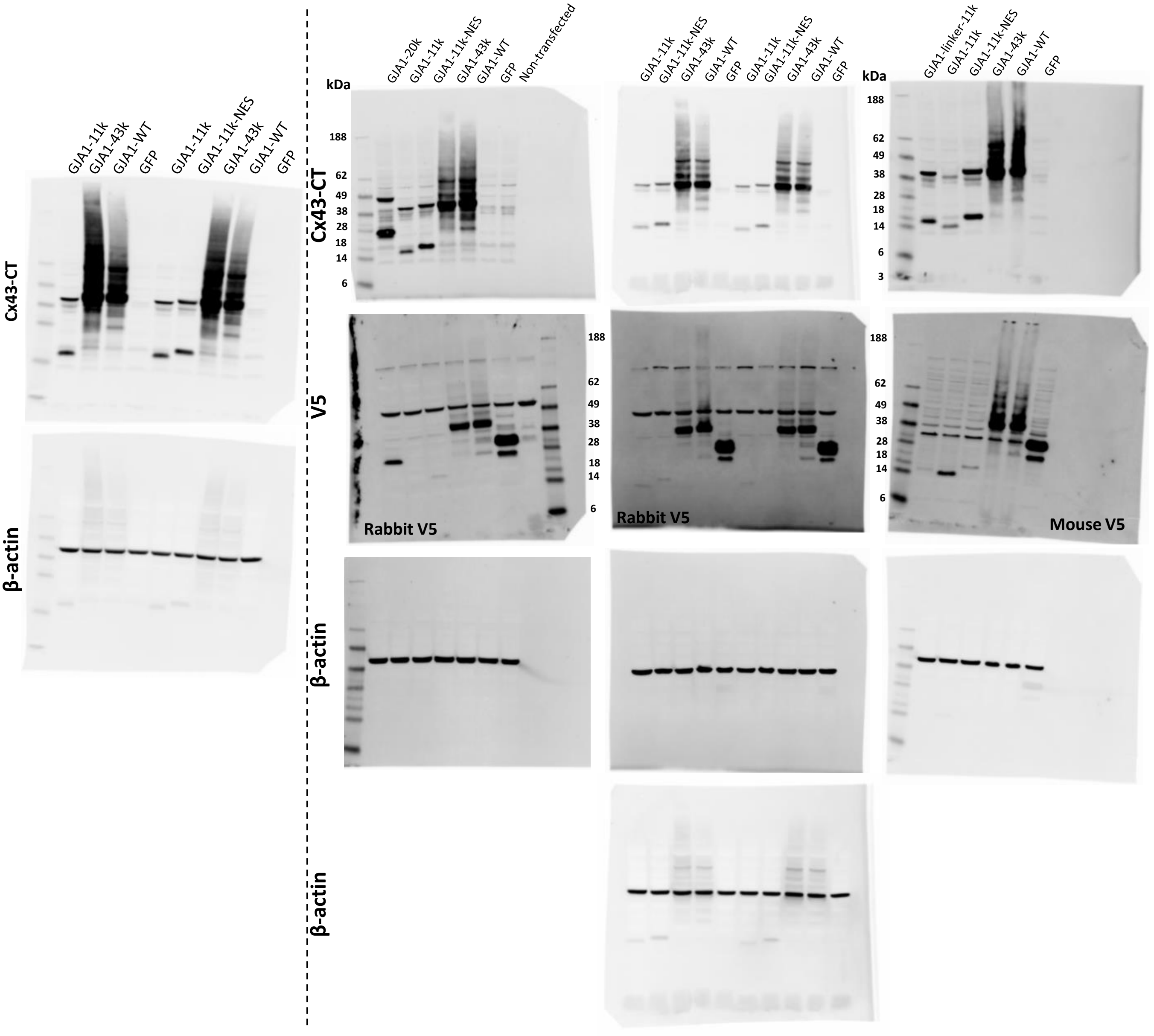

Figure S6: Uncropped immunoblots for Figure 4 and Figure 5 probed to Cx43 and V5 antibodies.

Supplemental Figure 7

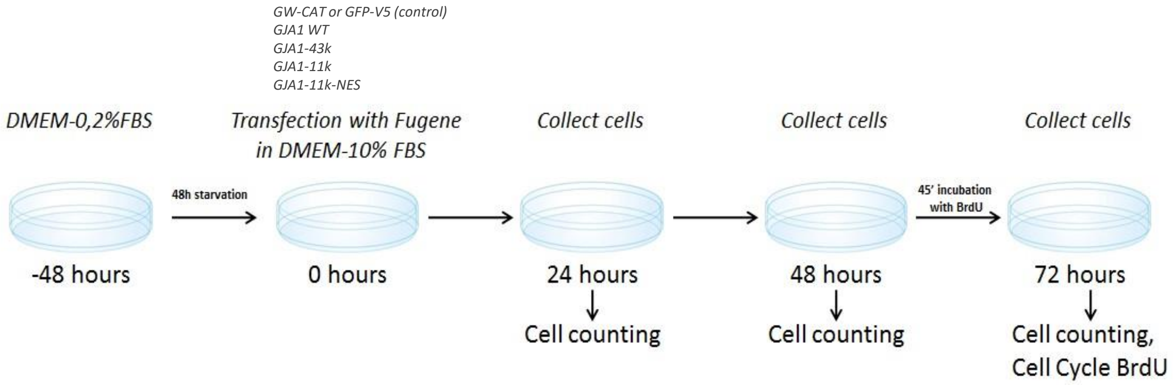

Figure S7: Schematic of the cell synchronization protocol adopted for cell count and cell cycle experiment.
